# *QuickStats:* Age-Adjusted Percentage* of Adults Aged ≥18 Years Who Had a Repetitive Strain Injury During the Past 3 Months,^†^ by Sex and Race and Hispanic Origin^§^ — National Health Interview Survey, United States, 2021^¶^

**DOI:** 10.15585/mmwr.mm7219a5

**Published:** 2023-05-12

**Figure:**
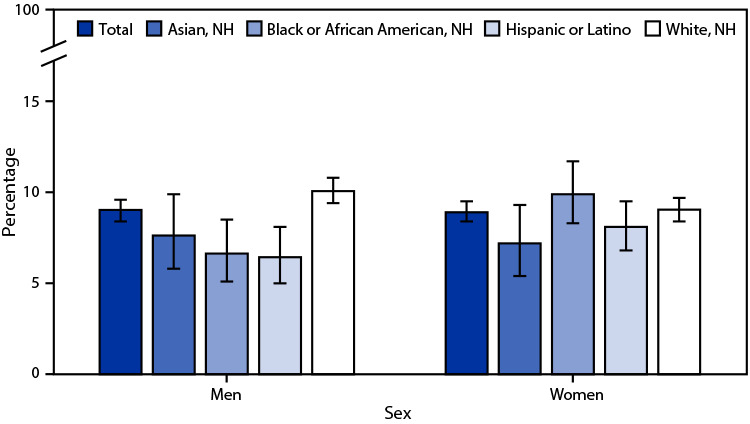
In 2021, 9.0% of men and 8.9% of women aged ≥18 years had a repetitive strain injury during the past 3 months. Non-Hispanic White men (10.1%) were more likely to have a repetitive strain injury than were non-Hispanic Asian (Asian) (7.6%), non-Hispanic Black or African American (Black) (6.6%), and Hispanic or Latino (Hispanic) (6.4%) men. Black women (9.9%) were more likely to have a repetitive strain injury than were Asian women (7.2%); there were no significant differences between other race and Hispanic origin groups for women. Among Black persons, men were less likely to have a repetitive strain injury than were women. Percentages of repetitive strain injuries among other race and Hispanic origin groups were similar between men and women.

**Source:** National Center for Health Statistics, National Health Interview Survey, 2021. https://www.cdc.gov/nchs/nhis/index.htm

